# Higher baseline inflammatory marker levels predict greater cognitive decline in older people with type 2 diabetes: year 10 follow-up of the Edinburgh Type 2 Diabetes Study

**DOI:** 10.1007/s00125-021-05634-w

**Published:** 2021-12-21

**Authors:** Anniek J. Sluiman, Stela McLachlan, Rachel B. Forster, Mark W. J. Strachan, Ian J. Deary, Jackie F. Price

**Affiliations:** 1 Usher Institute, University of Edinburgh, Edinburgh, UK; 2 Centre for Cognitive Ageing and Cognitive Epidemiology, University of Edinburgh, Edinburgh, UK; 3 Metabolic Unit, Western General Hospital, Edinburgh, UK; 4 Department of Psychology, University of Edinburgh, Edinburgh, UK

**Keywords:** Cognitive decline, C-reactive protein, Fibrinogen, Interleukin 6, Older people, Systemic inflammation, Type 2 diabetes

## Abstract

**Aims/hypothesis:**

We aimed to determine the longitudinal association of circulating markers of systemic inflammation with subsequent long-term cognitive change in older people with type 2 diabetes.

**Methods:**

The Edinburgh Type 2 Diabetes Study is a prospective cohort study of 1066 adults aged 60 to 75 years with type 2 diabetes. Baseline data included C-reactive protein, IL-6, TNF-α fibrinogen and neuropsychological testing on major cognitive domains. Cognitive testing was repeated after 10 years in 581 participants. A general cognitive ability score was derived from the battery of seven individual cognitive tests using principal component analysis. Linear regression was used to determine longitudinal associations between baseline inflammatory markers and cognitive outcomes at follow-up, with baseline cognitive test results included as covariables to model cognitive change over time.

**Results:**

Following adjustment for age, sex and baseline general cognitive ability, higher baseline fibrinogen and IL-6 were associated with greater decline in general cognitive ability (standardised *β*s = −0.059, *p*=0.032 and −0.064, *p*=0.018, respectively). These associations lost statistical significance after adjustment for baseline vascular and diabetes-related covariables. When assessing associations with individual cognitive tests, higher IL-6 was associated with greater decline in tests of executive function and abstract reasoning (standardised *β*s = 0.095, *p*=0.006 and −0.127, *p*=0.001, respectively). Similarly, raised fibrinogen and C-reactive protein levels were associated with greater decline in processing speed (standardised *β*s = −0.115, *p*=0.001 and −0.111, *p*=0.001, respectively). These associations remained statistically significant after adjustment for the diabetes- and vascular-related risk factors.

**Conclusions/interpretation:**

Higher baseline levels of inflammatory markers, including plasma IL-6, fibrinogen and C-reactive protein, were associated with subsequent cognitive decline in older people with type 2 diabetes. At least some of this association appeared to be specific to certain cognitive domains and to be independent of vascular and diabetes-related risk factors.

**Graphical abstract:**

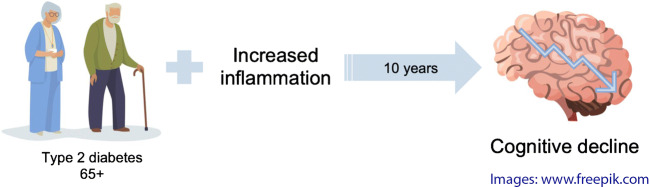

**Supplementary Information:**

The online version contains peer-reviewed but unedited supplementary material available at 10.1007/s00125-021-05634-w.

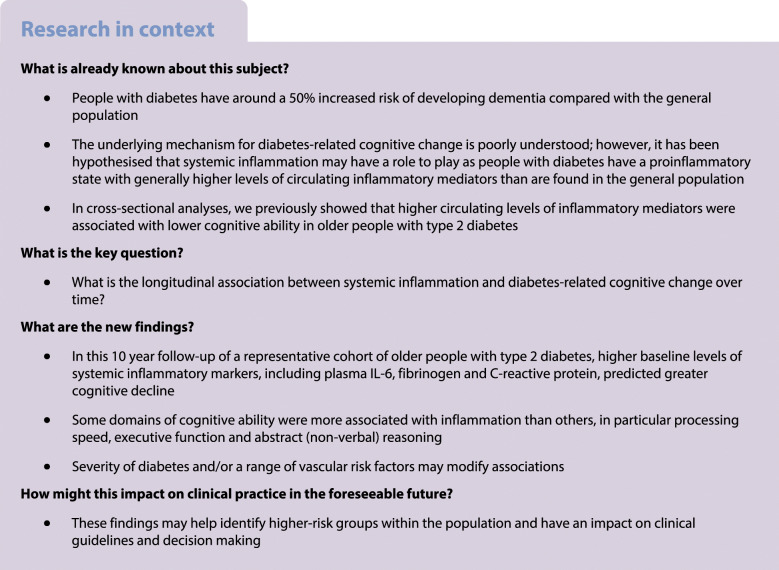



## Introduction

Diabetes mellitus, recognised as a major public health challenge [1], is ranked as the fourth leading cause of disability worldwide and is predicted to be the seventh leading cause of death in 2040 [2]. In addition to well-recognised cardiovascular and renal complications of diabetes, people with diabetes have around a 50% increased risk of developing dementia compared with the general population [3]. This includes both Alzheimer’s disease and vascular dementia (relative risks 1.46 and 2.48, respectively) [4]. Worldwide, 826,000 cases of Alzheimer’s disease are attributable to diabetes, resulting in a population attributable risk of 2.4% [5]. Diabetes is also associated with cognitive impairment that falls short of dementia [6], and with accelerated rates of age-related cognitive decline [7], resulting in earlier age of onset of clinically relevant cognitive impairment by 2.5 years [8].

Diabetes-related cognitive change is likely the result of multiple pathophysiological processes including vascular lesions, loss of white matter and general brain atrophy [8]; however, as yet, the underlying mechanism is poorly understood [9]. Rat models have shown that cerebral insulin levels may play a role in Alzheimer-like pathologies [10], while clinical studies have shown that people with diabetes-related dementia are more likely to have vascular dementia pathology [11]. A number of key vascular risk factors have been identified including impaired fasting glucose and insulin [12, 13], hypertension and macrovascular disease [14, 15]. It has also been hypothesised that systemic inflammation may have a role to play as people with diabetes have a proinflammatory state, with generally higher levels of circulating inflammatory mediators than are found in the general population [16]. Moreover, it has been found that low-grade inflammation predicts incident type 2 diabetes, with the inflammatory marker IL-6 driving this association [17].

In the general population, there is increasing evidence that inflammatory markers may predict cognitive change [18], and, in cross-sectional analyses, we showed previously that higher circulating levels of inflammatory mediators were associated with lower cognitive ability in older people with type 2 diabetes [19]. However, to date, little is known about the longitudinal association between systemic inflammation and diabetes-related cognitive change over time. In this 10 year follow-up study from the Edinburgh Type 2 Diabetes Study (ET2DS), we aimed to determine the longitudinal association of specific circulating markers of systemic inflammation measured at baseline with subsequent long-term cognitive change in older people with type 2 diabetes.

## Methods

### Study population

The ET2DS is a population-based prospective cohort study set up in 2006/2007 of 1066 men and women aged between 60 and 75 years at baseline with type 2 diabetes, living in Edinburgh and the Lothians. Details of recruitment have been published previously [20], but, in brief, participants were recruited at random, selected within sex and 5 year age bands from the Lothian Diabetes Register, a database with information on around 20,000 people diagnosed with diabetes according to WHO criteria. Inclusion criteria included being on oral glucose-lowering medications and/or insulin. If diabetes was managed through diet control alone, an HbA_1c_ measure of >48 mmol/mol (6.5%) at the baseline clinic was required. Medical records were consulted if diabetes status of a potential participant was unclear.

The final study population of 1066 people has been shown previously to be largely representative of the target population of all older people with type 2 diabetes living in Lothian, Scotland, in terms of a range of sociodemographic and clinical characteristics [19]. The baseline population allows for 90% power at the two-sided 5% significance level to detect correlation of ≥0.1 of two continuous variables.

Participants were followed up for 10 years, including three intensive phases of data collection at years 1, 4 and 10 after recruitment. Other than those known to have died, all original participants were invited to re-attend the year 10 follow-up. Participants were invited to attend a research clinic, or, at year 10 follow-up, were visited at home by the research team if unable to attend a clinic. To further maximise attendance rates, participants were offered travel expenses to the clinic and a range of possible dates and times for clinics or home visits, and multiple attempts of contact were made by both post and telephone.

Ethical approval was granted from the Lothian Research Ethics Committee and NHS Lothian Research and Development Office. Written informed consent was obtained from all participants on attendance at each clinical and cognitive examination phase of the study.

### Physical examination

Details of the physical and cognitive examination undertaken at baseline, including a fasting venous blood sample and a self-completion questionnaire on past medical history, together with record linkage to hospital discharge data, have been described previously [20]. Data collection took place at the Wellcome Trust Clinical Research Facility at the Western General Hospital, Edinburgh and record linkage was undertaken via the Information and Statistics Division of the National Health Service in Scotland. Baseline data were collected on age; sex; educational attainment levels; duration of diabetes; medications; smoking and alcohol history; past cardiovascular events and diabetic retinopathy; plasma HbA_1c_ and glucose; serum total cholesterol, triacylglycerols and HDL-cholesterol; systolic and diastolic blood pressure; and BMI.

### Measurement of inflammatory markers

At baseline, fasting blood samples were processed at the research clinic for immediate measurement of C-reactive protein (CRP), IL-6, TNF-α and fibrinogen. CRP was assayed using a high-sensitivity immunonephelometric assay (Dade Behring, UK) and TNF-α and IL-6 antigen levels were determined using high-sensitivity ELISA kits (R&D Systems, UK), performed in the University Department of Medicine, Glasgow Royal Infirmary. Participants with CRP level above 10 mg/l were removed from analyses, as this is indicative of acute inflammation likely as a result of a temporary infection and not chronic low-level inflammation.

### Cognitive assessment

The same cognitive test battery of seven neuropsychological tests was undertaken at baseline and at 10 year follow-up. Verbal and non-verbal memory was measured using the Logical Memory (LM) and Faces subtests of the Wechsler Memory Scale, Third Edition (UK), respectively [21]. Executive function was tested using the Borkowski Verbal Fluency Test (BVFT) and the Trail Making Test B (TMTB). Processing speed, working memory and abstract (non-verbal) reasoning were assessed using the Digit Symbol Test (DST), Letter–Number Sequencing (LNS) and Matrix Reasoning (MR) subtests of the Wechsler Adult Intelligence Scale, Third Edition (UK), respectively [22]. This test battery was selected for the cohort at baseline to capture the major cognitive domains thought to be susceptible to decline in people with diabetes [20]. Following a test of sufficient visual acuity and a blood glucose level of above 4 mmol/l, the participants undertook the cognitive examination, which lasted no more than 1 h. The use of visual and/or hearing aids was encouraged, if required. The Hospital Anxiety and Depression Scale (HADS) [23] was used to measured self-reported symptoms of depression and anxiety on a scale of 0–21.

### Statistical analysis

#### General cognitive function

In order to establish a measure of general cognitive ability for each participant, a principal component analysis (PCA) was performed using age-adjusted cognitive data from the seven cognitive tests at baseline (LM, Faces, TMTB, MR, DST, BVFT and LNS) using an extraction of Eigenvalues of >1.

Based on the approach of Gow et al [24], all cognitive data at both time points were stacked into seven columns and a single component of general cognitive ability was determined. Regression scores of the first component, termed general cognitive ability, ‘*g*’, were then saved according to time point, allowing baseline ‘*g*’ values to be centred on zero with an SD of 1 and follow-up ‘*g*’ values to be relative to baseline scores. Ten year cognitive change was represented by adjustment of year 10 scores for baseline scores. This method is now well established and is preferable to raw change scores as it is less dependent on individual differences in initial cognitive status [25].

PCA is only possible for cases with full cognitive data at baseline and at follow-up. In order to allow as many cases to be included in analyses as possible and to ensure sufficient power of analyses of variable ‘*g*’, multiple imputation was used for this variable [26]. To impute missing values a number of likely values based on the age, sex and other cognitive test results of the case were generated and a mean was taken. Data were assumed not to be missing at random, as some participants had a physical disability that prevented some cognitive tests being carried out, while others may not have been cognitively able to fully understand the instructions of the task. To ensure test scores were not artificially inflated as a result of imputation, imputations were only carried out on data where there were fewer than three out of seven cognitive tests missing. It should be noted that all other data described and analysed, other than the latent general cognitive function variable ‘*g*’, are on non-imputed data.

#### Analysis

Linear multivariable regression analyses were used to determine longitudinal associations between baseline risk factors and cognitive outcomes. A similar approach was used to determine the cross-sectional associations between year 10 risk factors and cognitive abilities. All models adopted a hierarchical approach, whereby blocks of diabetes-related (duration of diabetes, HbA_1c_ and medication status) and cardiovascular risk factors (hypertension, smoking, HDL-cholesterol, serum triacylglycerols, alcohol units, and anxiety and depression scores) were sequentially added to the models to explore the association of each predictor risk factor and cognitive outcome. To determine the association of a risk factor with cognitive change over time, cognitive outcomes at year 10 were adjusted for baseline cognitive performance [24]. Additionally, categorisation of cognitive outcomes allowed for the calculation of ORs for ‘accelerated’ 10 year cognitive decline (lowest vs highest tertile of follow-up ‘*g*’ adjusted for baseline ‘*g*’) using logistic regression. All analyses were carried out in SPSS version 21 [27].

## Results

**Fig. 1:**
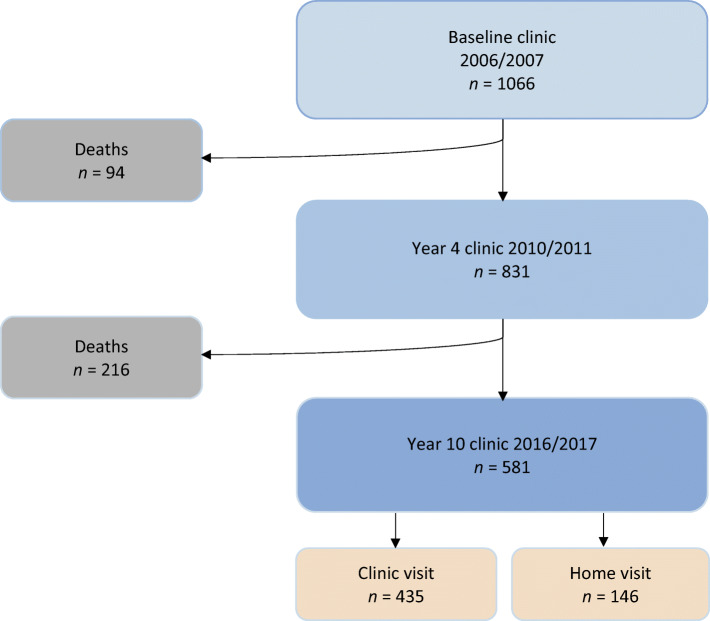
Follow-up for cognitive testing in the ET2DS

The baseline characteristics of the ET2DS cohort and the characteristics of those who re-attended at the year 10 follow-up are described in Table 1. A total of 581 participants returned for cognitive testing after 10 years, which represented 77% of those participants still living (*n* = 756) at the time of invitation for repeat testing; 25% were tested at a home visit (see Fig. 1). The attrition rate over the full 10 years of the study was 45.5% (*n* = 485). The most common reason for non-attendance was death of participant (*n* = 310), accounting for 29.1% of the total baseline study population. The second highest reason given for non-attendance was comorbidities preventing attendance (*n* = 80), accounting for 7.5% of the total baseline study population (see Table 2). A comparison between baseline characteristics of attenders and non-attenders of the ET2DS at year 10 is presented in electronic supplementary material (ESM) Table 1.

**Table 1:** Baseline characteristics of ET2DS population and of participants attending for repeat cognitive testing after 10 years

Characteristic	ET2DS (*N* = 1066)	10 year attenders (*n* = 581)	Missing data at baseline (%)
Age (years)	67.9 ± 4.2	67.3 ± 4.2	0
Sex (male)	547 (51.3)	296 (50.9)	0
Duration of diabetes (years)	8.1 ± 6.5	8.2 ± 9.8	1.21
HbA_1c_ (mmol/mol)	57.4 ± 12.3	57.4 ± 12.3	0.94
HbA_1c_ (%)	7.4 ± 1.1	7.4 ± 1.1	0.94
Medication			
Insulin (+/− oral hypoglycaemic agents)	186 (17.4)	89 (15.3)	0
Oral hypoglycaemic agents	681 (63.9)	365 (62.8)	0
Diet controlled	199 (18.7)	127 (21.9)	0
Plasma glucose (mmol/l)	7.6 ± 2.1	7.5 ± 1.9	0
Systolic blood pressure (mmHg)	133.3 ± 16.4	132.1 ± 14.7	1.59
Diastolic blood pressure (mmHg)	69.1 ± 9.0	69.4 ± 8.5	0.19
Plasma HDL-cholesterol (mmol/l)	1.29 ± 0.36	1.31 ± 0.40	0.19
Serum triacylglycerols (mmol/l)	1.70 ± 0.65	1.69 ± 0.64	0.84
Total cholesterol (mmol/l)	4.3 ± 0.9	4.3 ± 0.9	0.75
BMI (kg/m^2^)	31.4 ± 5.7	31.1 ± 5.5	0
Alcohol units	9.01 ± 14.48	9.85 ± 14.5	0
Smoking status			0
Current smoker	154 (14.4)	70 (12.0)	0
Ex-smoker	414 (38.8)	246 (42.3)	0
Never smoked	498 (46.7)	265 (45.6)	2.06
Stroke/TIA (yes)	93 (8.7)	44 (7.6)	0
Coronary heart disease (yes)	330 (31.0)	150 (25.8)	0
Diabetic retinopathy (yes)	339 (32.5)	173 (30.1)	0
Education			
Primary school	7 (0.7)	3 (0.5)	0
Secondary school	581 (54.4)	300 (51.6)	0
Professional qualification	307 (28.8)	169 (29.1)	0
University/college	171 (16.0)	109 (18.8)	0
MMSE <24	47 (4.4)	18 (3.1)	0.28
HADS	3.9 ± 2.9	3.5 ± 2.7	0.09
Inflammatory markers			
CRP (mg/l)	1.86 (0.87–4.37)	1.61 (0.79–3.65)	2.25
IL-6 (pg/ml)	2.87 (1.97–4.46)	2.56 (1.77–8.81)	0.19
TNF-α (pg/ml)	1.07 (0.69–1.62)	1.02 (0.64–1.44)	0.28
Fibrinogen (ng/ml)	3.6 (3.14–4.10)	3.7 (3.18–4.21)	0.28
Cognitive tests			
LM	25.2 ± 8.2	25.8 ± 8.1	1.50
DST	49.2 ± 14.8	52.4 ± 14.4	0.84
TMTB	104 (81–138)	98 (77–127)	1.31
Faces	65.8 ± 7.9	67.0 ± 7.8	0.66
LNS	9.7 ± 2.8	10.1 ± 2.7	1.69
MR	12.8 ± 5.3	13.8 ± 5.3	1.31
BVFT	36.9 ± 12.8	38.4 ± 12.6	0.56
*g*	0.00 ± 1.00	0.3 ± 0.9	0.47

Data are mean ± SD, median (IQR) or *n* (%)

ET2DS, max *n* = 1066, min *n* = 1042; 10 year attenders, max *n* = 581, min *n* = 569

TIA, transient ischaemic attack

**Table 2:** Reasons for non-attendance at year 10 follow-up

Principle reason for non-attendance^a^	Attrition at year 10 follow-up (*n*)	% of non-attenders (total *n* = 485)
Health concerns	80	16.49
Full-time carer	10	2.06
Relocation	8	1.65
Withdrawn	53	10.93
Non-contactable	24	4.95
Deceased	310	63.92

^a^Reasons given by participant, participant’s representative or medical report

**Fig. 2:**
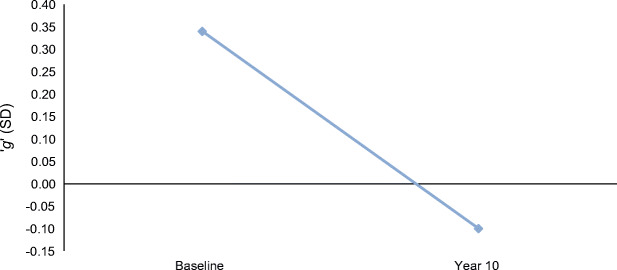
The decline in general cognition (‘*g*’) between baseline and year 10 for those who attended follow-up (*n* = 581). Data are reported as mean SD units

Table 3 describes the baseline and follow-up cognitive test scores of the participants who attended the year 10 follow-up. Average performance on cognitive tests declined over the 10 years, including TMTB (where a higher score is indicative of a poorer performance). Mean ‘*g*’ at baseline for the ET2DS (*N* = 1066) was standardised to 0.00 ± 1.00. The population who re-attended at year 10 had a higher baseline general cognition score than the population as a whole had at baseline (0.34 ± 0.85); however, despite this, they showed a marked decline over the course of the study. In terms of effect size, ‘*g*’ declined by a considerable 0.44 SD (see Fig. 2). At year 10 follow-up, 52 participants scored <24 on the Mini Mental State Exam (MMSE).

**Table 3:** Baseline and follow-up cognitive test scores of 10 year attenders

Cognitive test	*n*	Baseline score of population attending follow-up (mean ± SD)	Year 10 follow-up score of population attending follow-up (mean ± SD)
*g*	526	0.34 ± 0.85	−0.10 ± 0.90
LM	577	25.83 ± 8.06	23.32 ± 9.21
TMTB^a^	544	97 (76–124)^a^	127 (98–178)^a^
Faces	563	66.99 ± 7.83	67.86 ± 8.75
MR	568	13.77 ± 5.32	10.96 ± 5.32
DST	553	52.37 ± 14.39	43.90 ± 14.28
BVFT	576	38.37 ± 12.62	35.50 ± 13.29
LNS	567	10.09 ± 2.69	7.73 ± 3.31

^a^Median (IQR)

The associations between the inflammatory markers and cognitive outcomes as measured by ‘*g*’ are presented in Table 4 (associations between inflammatory markers and the individual cognitive tests are described in ESM Table 2). Cognitive ability at follow-up was associated, after adjustment for age and sex, with a number of the inflammatory markers. The majority of statistically significant associations between inflammatory markers and either individual cognitive tests or ‘*g*’ were seen for IL-6, followed by fibrinogen and CRP. The strongest and most numerous associations with individual tests (with βs typically between 0.1 and 0.2) were with DST and TMTB, which assess processing speed, and also with MR, which assesses abstract reasoning. There were few associations with memory-based tests.

**Table 4:** Association between baseline inflammatory markers and cognitive decline as measured by individual cognitive tests and ‘*g*’ at year 10 follow-up

Inflammation marker	*g* Standardised β (SE)
Fibrinogen	
+ age and sex	−0.111** (0.048)
+ age, sex and baseline cognition score	−0.059* (0.031)
+ baseline diabetes covariates	−0.049 (0.031)
+ baseline cardiovascular covariates	−0.035 (0.032)
lnCRP	
+ age and sex	−0.101* (0.045)
+ age, sex and baseline cognition score	−0.055 (0.029)
+ baseline diabetes covariates	−0.045 (0.029)
+ baseline cardiovascular covariates	−0.029 (0.030)
lnIL-6	
+ age and sex	−0.152** (0.042)
+ age, sex and baseline cognition score	−0.064* (0.028)
+ baseline diabetes covariates	−0.052 (0.028)
+ baseline cardiovascular covariates	−0.039 (0.029)
lnTNF-α	
+ age and sex	−0.055 (0.042)
+ age, sex and baseline cognition score	−0.023 (0.027)
+ baseline diabetes covariates	−0.015 (0.027)
+ baseline cardiovascular covariates	−0.015 (0.027)

Data for ‘*g*’ (general cognitive ability) have been imputed. Diabetes covariables: duration of diabetes, HbA_1c_ and medication status. Cardiovascular covariables: hypertension, smoking, HDL-cholesterol, serum triacylglycerols, alcohol units, and anxiety and depression scores

*p<0.05

**p<0.01

The association with cognitive change over time was assessed by adjusting for age, sex and baseline ‘*g*’. Higher fibrinogen (standardised *β* −0.059, *p*=0.032) and IL-6 (standardised *β* −0.064, *p*=0.018) were significantly associated with a decline in general cognitive ability. Higher CRP, fibrinogen and IL-6 were significantly associated with the individual cognitive tests, including TMTB (IL-6 standardised *β* 0.095, *p*=0.006), MR (IL-6 standardised *β* −0.127, *p*=0.001) and DST (CRP standardised *β* −0.111, *p*=0.001; fibrinogen standardised *β* −0.115, *p*=0.001).

In addition to the adjustments for age, sex and baseline cognition, further adjustment for a range of diabetes- and vascular-related risk factors reduced the strength and statistical significance of the findings. However, after adjustment for specific diabetes-related risk factors (duration of diabetes, HbA_1c_ and medication status as a measure of disease severity), higher IL-6 was still significantly associated with a decline in cognitive ability as measured by DST (standardised *β* −0.076, *p*=0.02), MR (standardised *β* −0.119, *p*<0.001) and TMTB (standardised *β* 0.089, *p*=0.01). Similarly, higher fibrinogen was significantly associated with a decline in cognitive ability as measured by DST (standardised *β* −0.107, *p*=0.001) and TMTB (standardised *β* −0.082, *p*=0.02), and higher CRP and TNF-α were significantly associated with cognitive decline as measured by DST (standardised *β*s −0.099, *p*=0.03; and −0.073, *p*=0.02, respectively).

When cardiovascular risk factors, including hypertension, smoking, HDL-cholesterol, serum triacylglycerols, alcohol, and anxiety and depression scores, were further adjusted for, higher IL-6 remained associated with TMTB (standardised *β* −0.076, *p*=0.04) and MR (standardised *β* −0.111, *p*=0.001), and fibrinogen, CRP and TNF-α remained associated with DST (standardised *β*s −0.086, *p*=0.008; −0.087, *p*=0.009; −0.083, p=0.008, respectively). Associations with ‘*g*’ did not remain statistically significant (data presented in ESM Table 2).

The association between IL-6 and fibrinogen was modelled against 10 year cognitive change, as scoring in the lowest tertile of year 10 ‘*g*’ adjusted for baseline ‘*g*’. Cut points for follow-up ‘*g*’ adjusted for baseline ‘*g*’: lowest tertile, <−0.16; medium tertile, −0.15 to 0.33; highest tertile, >0.34.

In fully adjusted models for age, sex and all diabetes and cardiovascular risk factors, each increase in IL-6 per SD was associated with 64% increased odds of accelerated 10 year cognitive decline (OR 1.64, *p* = 0.006), and each increase per SD in fibrinogen was associated with 69% increased odds of accelerated 10 year cognitive decline (OR 1.69, *p*=0.001).

## Discussion

In this 10 year follow-up of a representative cohort of older people with type 2 diabetes, higher baseline levels of systemic inflammatory markers, including plasma IL-6 and fibrinogen, predicted greater cognitive decline. This primary finding of our research involved association with a single test of generalised cognitive ability, derived from multiple individual cognitive tests that are specific to certain cognitive domains. We also found associations between individual cognitive tests and some of the inflammatory markers. In this respect, processing speed and executive function (measured using the TMTB and the DST), and abstract reasoning (measured using the MR), appeared to be particularly associated with altered inflammation. Our results also indicated that severity of diabetes and/or a range of vascular risk factors modified associations to a greater or lesser extent, depending on the inflammatory marker and the cognitive test. This suggests that inflammation may play a differential role across specific cognitive functions, but that at least some effects may be independent of conventional vascular risk factors known to contribute to cognitive dysfunction during ageing. Effect sizes overall were modest, though statistically significant, when the outcome was considered as a continuous outcome. However, we also showed that altered inflammatory marker levels were associated with a moderate increase in odds of being in a more severe tertile of cognitive decline, a potentially more clinically relevant and interpretable measure of age-related cognitive dysfunction.

Previously, in the baseline phase of the ET2DS, we showed associations between raised plasma inflammatory markers (including IL-6 and TNF-α) and lower cognitive ability [19]. Since this time, a number of studies have also found similar cross-sectional associations, mainly in the general population [28], but also in people with type 2 diabetes [18]. However, the important question of whether differences in inflammatory markers might precede the development of cognitive impairment, i.e. that inflammatory markers are associated with subsequent cognitive decline, has remained largely unanswered, especially for high-risk populations such as people with type 2 diabetes. Our current results are consistent with the hypothesis that raised levels of inflammation precede accelerated cognitive decline. This is also in line with emerging evidence presented from studies in the general population [28] and on frank dementia, where a recent systematic review has shown that increased levels of systemic inflammatory markers, namely IL-6, fibrinogen and CRP, increased the risk of developing all-cause dementia, though not with Alzheimer’s disease alone [29]. Our results build on previous work by first exploring cognitive change, as opposed to frank dementia, as this provides a more nuanced exploration of the association with inflammation, taking into account individual differences and more subtle changes in cognition. Second, we explore this association specifically in people with type 2 diabetes who are known to have an increased risk of developing both systemic inflammation and cognitive decline.

There are limitations to consider in our analyses. Over the 10 year follow-up period, there was an inevitable number of drop-outs from the ET2DS; however, the retention rate (55%) was in line with other studies [30] and relatively high when taking into account both participant age [31] and diabetes status [32]. Considerable effort, including the offer of transport or reimbursement of transport costs to and from the clinic, as well as the offer of a home visit, was made by the study team to ensure that as many of the surviving original cohort as possible were seen at year 10. It should also be recognised that any findings reported on associations between predictors and outcomes make no assumption on causality, despite the insights into temporality provided by the prospective study design and which suggest that reverse causation is unlikely to be a major explanation for our findings. We were unable to assess change in inflammatory marker levels over time in relation to the cognitive changes, and this would be relevant to consider in future studies. Fluctuations in levels of circulating inflammatory markers in individual cases may have occurred over time, including in response to lifestyle factors, illness and the use of medication. These latter factors themselves may also be linked both to baseline inflammatory marker levels and with a greater rate of cognitive change during the follow-up period, potentially helping to explain our findings. Despite these limitations, it should be noted that levels of inflammatory markers at baseline did predict subsequent cognitive change, and that this association was independent of a wide range of potentially confounding baseline biological risk factors. The ET2DS population at baseline exclusively consists of people with type 2 diabetes who have survived until the age of 65 years and so can be assumed to be a healthier subgroup of all people with type 2 diabetes. In addition, these analyses represent cognitive data from participants still living at follow-up. Survivor bias therefore plays a role within this research and should be taken into account as estimates will lie closer to the null, with a smaller effect size than likely observed in the true population.

In summary, we provide evidence for an association between increased levels of systemic inflammation as measured at baseline and subsequent 10 year cognitive decline in people with type 2 diabetes. IL-6 and fibrinogen were significantly associated with a decline in general cognition, although in fully adjusted models for a wide range of potential confounding and/or mediating factors, these associations were no longer statistically significant. Conversely, individual cognitive tests (TMTB, DST and MR) remained significantly associated with IL-6 and/or fibrinogen. Although these inflammatory markers are known to be highly correlated, it is possible that specific markers differ in their strength of association with cognitive decline, and also with decline within different domains of cognition. Furthermore, some of the associations may be mediated by vascular and diabetes-related risk factors. Any biological effect of inflammatory markers on cognition can only be surmised from the results of the current study, although the inflammatory marker IL-6 is known to cross the blood–brain barrier and so could have either a direct or indirect intracerebral effect on cognition [33]. Future studies in this area could usefully assess the amount of variance explained by inflammation and consider a wider range of individual and multiple covariables, change in covariables over time and inter-relations between covariables.

Our results go some way to addressing the previous paucity of evidence of a prospective association between markers of systemic inflammation and cognitive decline in people with type 2 diabetes. As this is primarily an exploratory observational study, clinical implications are limited and indirect. For example, while our results would be consistent with a true pathophysiological association, further investigation is required to determine whether associations are causal. If this can be proven, the possibility arises that efforts taken to reduce overall systemic inflammation may be beneficial in preventing the onset and severity of cognitive decline in people with type 2 diabetes. In addition, even if the findings do not translate into direct impact on therapeutic pathways, they could potentially help identify higher-risk groups within the diabetes population and so impact on clinical guidelines and decision making.

## Supplementary Information

ESM (PDF 189 kb)

## Data Availability

The data that support the findings of this study are not publicly available due to them containing information that could compromise research participant privacy/consent. Aggregate data may be made available from the corresponding author upon reasonable request.

## Abbreviations

BVFT: Borkowski Verbal Fluency Test
CRP: C-reactive protein
DST: Digit Symbol Test
ET2DS: Edinburgh Type 2 Diabetes Study
HADS: Hospital Anxiety and Depression Scale
LM: Logical Memory
LNS: Letter–Number Sequencing
MMSE: Mini Mental State Exam
MR: Matrix Reasoning
PCA: Principal component analysis
TMTB: Trail Making Test B
