# Supplementary material for: Higher baseline inflammatory marker levels predict greater cognitive decline in older people with type 2 diabetes: year 10 follow-up of the Edinburgh Type 2 Diabetes Study
Source: Diabetologia. 2021 Dec 21;65(3):467–76. doi: 10.1007/s00125-021-05634-w (PMC8803673; doi:10.1007/s00125-021-05634-w)
Supplement: Supplementary file 1 — (PDF 189 kb) [file 125_2021_5634_MOESM1_ESM.pdf]

Electronic Supplementary Material (ESM)

# ESM Table 1. Baseline characteristics of attenders and non-attenders of the ET2DS at Year 10

| Characteristic | Attenders | | Non-Attenders | | T or $X^2$ (p-value) |
|---|---|---|---|---|---|
| | N | Mean ± SD, median (IQR) or n (%) | N | Mean ± SD, median (IQR) or n (%) | |
| Demographic: | | | | | |
| Age (years) | 581 | 67.31 ± 4.2 | 485 | 68.62 ± 4.2 | -5.077 (<0.001) |
| Sex males (n %) | 581 | 296 (50.9) | 485 | 251 (51.8) | 0.069 (0.793) |
| SIMD rank: | 581 | | 485 | | 14.725 (0.005) |
| 1st quintile | | 56 (9.6) | | 71 (14.6) | |
| 2nd quintile | | 116 (20.0) | | 92 (19.0) | |
| 3rd quintile | | 92 (15.8) | | 96 (19.8) | |
| 4th quintile | | 103 (17.7) | | 91 (18.8) | |
| 5th quintile | | 214 (36.8) | | 135 (27.8) | |
| Educational attainment: | 581 | | 485 | | 8.234 (0.041) |
| University/ college | | 109 (18.8) | | 62 (12.8) | |
| Professional/ technical | | 169 (29.1) | | 138 (28.5) | |
| Secondary school | | 300 (51.6) | | 281 (57.9) | |
| Primary School | | 3 (0.5) | | 4 (0.8) | |
| Employment status: | 581 | | 485 | | 21.438 (0.001) |
| Full-time | | 47 (8.1) | | 28 (5.8) | |
| Part-time | | 54 (9.3) | | 23 (4.7) | |
| Unemployed | | 7 (1.2) | | 2 (0.4) | |
| Retired | | 443 (76.2) | | 421 (86.8) | |
| Homemaker | | 15 (2.6) | | 4 (0.8) | |
| Other | | 15 (2.6) | | 7 (1.4) | |
| Vascular related: | | | | | |
| Systolic blood pressure (mmHg) | 580 | 132.06 ± 14.7 | 484 | 134.79 ± 18.2 | -2.651 (0.008) |
| Diastolic blood pressure (mmHg) | 580 | 69.36 ± 8.5 | 484 | 68.69 ± 9.6 | 1.198 (0.231) |
| Hypertension (n) | 580 | 178 (30.7) | 484 | 189 (39.0) | 8.161 (0.004) |
| Total cholesterol (mmol/l) | 578 | 4.33 ± 0.9 | 479 | 4.29 ± 0.9 | 0.739 (0.462) |
| High density lipoprotein (mmol/l) | 578 | 1.31 ± 0.4 | 479 | 1.27 ± 0.4 | 1.975 (0.049) |
| Serum triglycerides (mmol/l) | 581 | 5.13 ± 58.5 | 485 | 14.04 ± 110.4 | -1.601 (0.110) |
| Retinopathy (n) | 575 | 173 (30.1) | 469 | 166 (35.4) | 3.318 (0.069) |
| Stroke (n) | 581 | 24 (4.1) | 485 | 38 (7.8) | 6.621 (0.010) |
| TIA (n) | 581 | 20 (3.4) | 485 | 11 (2.3) | 1.291 (0.256) |
| Smoking status (n) | 581 | | 485 | | 9.456 (0.009) |
| - Current | | 70 (12.0) | | 84 (17.3) | |

| | | | | | |
|---|---|---|---|---|---|
| - Former | | 246 (42.3) | | 168 (34.6) | |
| - Never | | 265 (45.6) | | 233 (48.0) | |
| Total cigarettes smoked | 577 | 1.66 ± 5.81 | 484 | 3.14 ± 8.02 | 6.874 (<0.001) |
| Alcohol units | 581 | 9.85 ± 14.5 | 485 | 8.00 ± 14.5 | 2.081 (0.038) |
| Diabetes related: | | | | | |
| Duration (median years (IQR)) | 581 | 8.19 ± 9.8 | 485 | 10.33 ± 13.9 | -2.857 (0.004) |
| HbA1c (median (IQR)) | 578 | 7.37 ± 1.1 | 479 | 7.45 ± 1.2 | -1.133 (0.258) |
| Plasma Glucose (mmol/L) | 574 | 7.48 ± 1.9 | 475 | 7.66 ± 2.3 | -1.406 (0.160) |
| Medication status (n) | 581 | | 485 | | 10.511 (0.005) |
| - Insulin ± oral | | 89 (15.3) | | 97 (20.0) | |
| - Oral | | 365 (62.8) | | 316 (65.2) | |
| - Diet controlled | | 127 (21.9) | | 72 (14.8) | |
| Obesity related: | | | | | |
| Body mass index (kg/m2) | 581 | 31.06 ± 5.5 | 484 | 31.86 ± 5.9 | -2.273 (0.023) |
| Waist circumference (cm) | 580 | 106.01 ± 12.7 | 481 | 107.95 ± 12.9 | -2.463 (0.014) |
| Waist to hip ratio | 580 | 0.96 ± 0.1 | 481 | 0.96 ± 0.1 | -1.004 (0.315) |
| Body fat (%) | 574 | 37.67 ± 7.7 | 478 | 38.60 ± 7.4 | -2.010 (0.045) |
| Inflammatory related: | | | | | |
| Fibrinogen (median ng/ml) | 580 | 3.57 ± 0.7 | 483 | 3.74 ± 0.8 | -3.677 (<0.001) |
| CRP (median mg/ml) | 569 | 3.15 ± 4.5 | 473 | 4.77 ± 7.4 | -4.161 (<0.001) |
| IL-6 (median pg/ml) | 580 | 3.45 ± 3.2 | 484 | 4.49 ± 3.8 | -4.771 (<0.001) |
| TNFα (median pg/ml) | 579 | 1.26 ± 1.7 | 484 | 1.47 ± 1.3 | -2.184 (0.029) |
| Psychological: | | | | | |
| MMSE <24 | 581 | 18 (3.1) | 485 | 29 (6.0) | 5.247 (0.022) |
| HADS A | 581 | 5.29 ± 3.7 | 484 | 6.24 ± 4.1 | -3.922 (<0.001) |
| HADS D | 581 | 3.47 ± 2.7 | 484 | 4.32 ± 3.0 | -4.785 (<0.001) |

Total attenders n = 581 (max). Total non-attenders n = 485 (max). Analysis is a two-tailed independent t-test or Pearson's chi squared. Values are means ± SD, median (interquartile range) or n (%). SIMD, Scottish Index of Multiple Deprivation; HbA1c, haemoglobin A1c; CRP, c-reactive protein; IL-6, interleukin-6; TNFα, tumour necrosis factor α; TIA, transient ischaemic attack; MMSE, Mini Mental State Exam; HADS A, Hospital Anxiety and Depression Scale-Anxiety subscale; Hospital HADS B, Hospital Anxiety and Depression Scale-Depression subscale.

ESM Table 2. Association between baseline inflammatory markers and cognitive decline as measured by individual cognitive tests and g at year 10

| Inflammation marker | g | LM | lnTMTB | Faces | MR | DST | BVFT | LNS |
|---|---|---|---|---|---|---|---|---|
| | Standardised Beta (SE) | | | | | | | |
| Fibrinogen<br>+ age and sex | -0.111** (0.048) | -0.024 (0.049) | 0.150*** (0.048) | -0.063 (0.049) | -0.099* (0.049) | -0.125** (0.049) | -0.067 (0.050) | -0.069 (0.049) |
| + baseline cognition score | -0.059* (0.031) | -0.001 (0.041) | 0.088* (0.040) | -0.036 (0.041) | -0.061 (0.038) | -0.115*** (0.036) | -0.052 (0.033) | -0.071 (0.043) |
| + baseline diabetes covariates | -0.049 (0.031) | 0.008 (0.041) | 0.082* (0.040) | -0.022 (0.041) | -0.051 (0.039) | -0.107*** (0.036) | -0.045 (0.033) | -0.061 (0.043) |
| + baseline cardiovascular covariates | -0.035 (0.032) | 0.034 (0.042) | 0.063 (0.041) | -0.006 (0.042) | -0.033 (0.040) | -0.086*** (0.037) | -0.032 (0.034) | -0.050 (0.044) |
| lnCRP<br>+ age and sex | -0.101* (0.045) | -0.027 (0.046) | 0.116** (0.046) | -0.031 (0.046) | -0.078 (0.046) | -0.137** (0.046) | -0.092* (0.047) | -0.058 (0.046) |
| + baseline cognition score | -0.055 (0.029) | -0.008 (0.039) | 0.072* (0.037) | -0.018 (0.038) | -0.028 (0.036) | -0.111** (0.034) | -0.056 (0.031) | -0.051 (0.041) |
| + baseline diabetes covariates | -0.045 (0.029) | 0.005 (0.040) | 0.065 (0.038) | -0.011 (0.039) | -0.023 (0.037) | -0.099** (0.034) | -0.049 (0.031) | -0.049 (0.041) |
| + baseline cardiovascular covariates | -0.029 (0.030) | 0.031 (0.041) | 0.050 (0.039) | 0.003 (0.040) | -0.010 (0.038) | -0.087** (0.035) | -0.035 (0.032) | -0.049 (0.0741) |
| lnIL-6<br>+ age and sex | -0.152** (0.042) | -0.096* (0.043) | 0.166*** (0.043) | -0.049 (0.043) | -0.176*** (0.043) | -0.154*** (0.044) | -0.080 (0.044) | -0.089* (0.044) |
| + baseline cognition score | -0.064* (0.028) | -0.069 (0.036) | 0.095** (0.036) | -0.013 (0.036) | -0.127*** (0.034) | -0.091** (0.033) | -0.017 (0.029) | -0.071 (0.038) |
| + baseline diabetes covariates | -0.052 (0.028) | -0.057 (0.037) | 0.089* (0.036) | 0.007 (0.037) | -0.119** (0.035) | -0.076* (0.033) | -0.011 (0.030) | -0.063 (0.039) |
| + baseline cardiovascular covariates | -0.039 (0.029) | -0.042 (0.038) | 0.076* (0.037) | 0.021 (0.038) | -0.111*** (0.036) | -0.061 (0.034) | 0.006 (0.031) | -0.052 (0.040) |
| lnTNF-α<br>+ age and sex | -0.055 (0.042) | -0.027 (0.042) | 0.083 (0.042) | -0.048 (0.042) | -0.053 (0.043) | -0.046 (0.042) | 0.004 (0.043) | -0.037 (0.043) |
| + baseline cognition score | -0.023 (0.027) | -0.023 (0.036) | 0.059 (0.035) | -0.035 (0.035) | -0.010 (0.034) | -0.084** (0.031) | 0.016 (0.028) | 0.001 (0.037) |
| + baseline diabetes covariates | -0.015 (0.027) | -0.014 (0.036) | 0.050 (0.035) | -0.023 (0.035) | -0.001 (0.034) | -0.073* (0.031) | 0.019 (0.029) | 0.008 (0.037) |
| + baseline cardiovascular covariates | -0.015 (0.027) | -0.018 (0.036) | 0.061 (0.035) | -0.024 (0.036) | -0.003 (0.034) | -0.083** (0.031) | 0.016 (0.029) | 0.014 (0.038) |

*p<0.05 **p<0.01 ***P<0.001.
Data for g (general cognitive ability) have been imputed, for remaining cognitive tests are non-imputed. Diabetes covariables; duration of diabetes, HbA1c and medication status.
Cardiovascular covariables; hypertension, smoking, HDL cholesterol, serum triglycerides, alcohol units, anxiety and depression scores.
LM, Logical Memory; MR, Matrix Reasoning; DST, Digit Symbol Test; lnTMTB, Trail Making Test B (natural log transformed); BVFT, Borkowski Verbal Fluency Test; CRP, c-reactive protein; IL-6, interleukin-6; TNF-α, tumour necrosis factor α.
